# Arsenic and smokeless tobacco induce genotoxicity, sperm abnormality as well as oxidative stress in mice *in vivo*

**DOI:** 10.1186/s41021-016-0031-2

**Published:** 2016-02-19

**Authors:** Samrat Das, Puja Upadhaya, Sarbani Giri

**Affiliations:** Department of Life Science and Bioinformatics, Molecular and Cell Biology Laboratory, Assam University, Silchar, 788011 India

**Keywords:** Sodium arsenite, Smokeless tobacco, Micronucleus assay, Sperm head abnormality, Lipid peroxidation, Reduced glutathione, Superoxide dismutase

## Abstract

**Background:**

Arsenic, a naturally occurring metalloid is a well-known water contaminant which causes a wide range of serious adverse health effects including cancer upon long-term exposure. Recent studies have shown high arsenic contamination in the ground water of North Eastern states of India including Southern Assam. Smokeless tobacco consumption locally known as “*sadagura*” is one of the most prevalent life style habit in southern Assam. The present study was undertaken in mice test system *in vivo*. Mice were exposed to smokeless tobacco (5 mg/kg body weight /day) and sodium arsenite (0.2 mg/kg body weight /day, 2 mg/kg body weight/day) independently and in combination for 90 days.

**Results:**

The results were compared with groups with only sodium arsenite exposure and groups which were exposed to only smokeless tobacco extract. Genotoxicity was evaluated by studying the incidence of micronucleated polychromatic erythrocytes from bone marrow. Both the tested doses of sodium arsenite induced statistically significant micronucleated polychromatic erythrocytes as compared to control group, however, sodium arsenite and smokeless tobacco extract could not increase the incidence of micronucleated polychromatic erythrocytes as compared to their individual counterparts when treated in combination in mice test system. Germ cell toxicity was evaluated by recording the sperm head abnormalities and total sperm count. Combined treatment of sodium arsenite and smokeless tobacco extract in lower dose induced a significant increase in sperm head abnormality as compared to only sodium arsenite and smokeless tobacco extract. Liver, kidney and intestine tissues were analyzed for various oxidative stress evaluations such as lipid peroxidation (MDA), Glutathione (GSH) and superoxide dismutase (SOD) assay. Sodium arsenite in combination with smokeless tobacco extract show higher genotoxic and germ cell toxic effects as compared to control but not when compared to their individual counterparts.

**Conclusion:**

Impairment of the sperm head morphology by sodium arsenite and smokeless tobacco extract alone and in combination with lower dose of sodium arsenite could be oxidative stress mediated effects. Besides, combination treatment of both the agents may not produce additive effects related to micronucleated polychromatic erythrocytes induction and decline of total sperm count.

## Background

Arsenic contamination of drinking water is a public health problem in South East Asia [1]. Approximately one-third of the hand tube wells in West Bengal and Bangladesh contain arsenic contaminated water [2]. Chronic exposure to arsenic has the potential to cause a wide range of carcinogenic and non-carcinogenic health effects such as cancer of the skin and internal organs, diabetes mellitus, hypertension and respiratory conditions [3–6]. Study on human colon cancer cells has shown that exposure to arsenic trioxide affects DNA synthesis and induce genotoxic effects [7]. Tobacco consumption may lead to increased risk of arsenic related skin lesions [8].

It has been reported that exposure to arsenic may reduce human semen quality [9]. Long-term exposure to arsenic have been reported to induce oxidative damage in human lymphocytes [10]. Studies have shown that ground water in many North Eastern states including Assam has been contaminated with arsenic [11]. High arsenic concentration has also been detected in the ground water of Barak valley region of Assam [12]. There are many region specific tobacco habits that are prevalent in India. *Sadagura*, a smokeless tobacco preparation is highly popular in Southern Assam. It consists of sun dried and roasted tobacco leaves with small amounts of black cumin and aniseed seeds as flavoring agents. Consumption of SG with or without areca nut often leads to increased incidence of micronucleus and other nuclear abnormalities in the buccal epithelial cells [13]. *Sadagura* extract alone and in combination with areca nut have been shown to cause genotoxic effects in mouse bone marrow cells [13]. Human cohort study has shown that the risk of stillbirth increases if smokeless tobacco is consumed during pregnancy [14]. Consumption of tobacco in any form may increase the chances of having oral cancer. The highest incidence of oral cancer in India is reported in Assam and the North Eastern region of India, where it is the second leading cancer among men and third among women [15]. Therefore, in the present study we evaluated the genotoxic potential of long-term exposure to sodium arsenite (SA) and *sadagura* extract either as single agent or in combination. Besides, we measured the oxidative stress induced by both the test agents and their combination.

Micronucleus (MN) assay of bone marrow cells is one of the fundamental assays by which the genotoxicity of a test substance can be determined [16]. Sperm head abnormality assay (SHA) is a reliable and widely used technique to determine the germ cell toxicity potential of test substances and is often used for mutagenicity and toxicological studies in mice test system [17–20]. Because of their essential role in metabolizing various toxic compounds in the body, oxidative stress evaluation was carried out in tissues of the liver, kidney and intestine. Lipid peroxidation assay relies on the measurement of level of malonaldehyde (MDA) in the tissue(s) which in turn is useful in estimating the extent of oxidative damage caused to the lipid integrity.

## Methods

### Test chemicals

Sodium arsenite solution (CAS No. 7784-46-5) was obtained from Sigma-Aldrich Laborchemikalien, Germany. NaCl, eosin (2 % w/v), Giemsa, EDTA, TCA, DTNB, 2-thiobarbituric acid and methanol were procured from HIMEDIA Laboratories, India. Tris HCl and pyragallol were purchased from Sisco Research Laboratories Pvt. Ltd, India. Stains and reagent solutions were prepared in double distilled water.

### Test animals, dosing and treatment

The study was approved by the Institutional Ethics Committee of Assam University, Silchar. Both male and female inbred strain of Balb/C mice (10–12 weeks; 20–25 g body weight) procured from Pasteur Institute, Shillong, Meghalaya, India were housed in laboratory conditions at room temperature of 25 ± 5 °C in photoperiod-controlled environment (12 L:12D cycles). The animals were provided with food pellets (Amrut Laboratory Animal Feeds, New Delhi) and water *ad libitum*. The animals were divided into six groups randomly (*n* = 6, 3 males and 3 females in each group). All the groups were exposed to their respective treatments for a period of 90 days. LD_50_ of sodium arsenite in mice is 15-22 mg /kg body weight. [21] We selected approximately 1 and 10 % of the reported LD_50_ doses for our study. A human equivalent dose was calculated for *sadagura* based on body surface area normalization method by Reagan-Shaw et al. [22]. The treatment groups consisted of (i) Group 1 (control), (ii) Group 2 (SA1, sodium arsenite- 0.2 mg/kg body weight/day), (iii) Group 3 (SA2, sodium arsenite- 2 mg/kg body weight/day), (iv) Group 4 (SG, *Sadagura* extract- 5gm/kg body weight/day), (v) Group 5 (SA1 + SG) and (vi) Group 6 (SA2 + SG). The test substances were given for a period of 90 days. The mode of administration of arsenic was through drinking water and aqueous extract of *sadagura* was given orally. Since s*adagura* is consumed orally as a chewing habit and arsenic exposure occurs through consumption of contaminated drinking water, the oral route was most relevant to assess toxicity of these agents.

### Preparation of s*adagura* extract

*Sadagura* extract was prepared as described earlier [23] with modifications. *Sadagura* was prepared as per the local formulation which consisted of dried tobacco leaves, aniseed and black cumin seeds mixed in 2:4:1 ratio and all the ingredients were purchased from local market in Silchar (Assam, India). The above ingredients were dried, roasted and grinded in a blender separately to a fine powder. The ingredients were mixed in the appropriate ratio and the mixture was continuously stirred in a shaker at 4 °C for 72 hrs. Subsequently, the mixture was filtered with cheesecloth to remove debris. The filtrate was then passed through Whatman filter paper No. 1 until clear. The extract was freeze dried in a lyophilizer. The crude extract was preserved in refrigerator at -20 ^0^C for not more than one month.

### Micronucleus assay

MN test in femur bone marrow cells were carried according to the method of Schmid [24] with minor modifications. Briefly, both the femur bones were flushed with 0.9 % NaCl (pre-warmed at 37 °C) instead of fetal calf serum. Smears of bone marrow cells were prepared in clean grease free slides, air-dried and fixed in absolute methanol for 10 min. The slides were then stained in 5 % Giemsa and mounted with cover slip using DPX mountant. The polychromatic erythrocytes (PCEs) stain light blue to gray and normochromatic erythrocytes (NCEs) stain light orange to straw yellow. At least 2000 PCEs were analyzed for the presence of MN. Number of NCEs per 2000 count of PCEs was recorded and PCE/NCE ratio was determined to assess the rate of erythropoiesis and cytotoxicity.

### Sperm head abnormality assay

The male germ cell toxicity study was carried out using sperm head abnormality (SHA) assay as described by Wyrobek and Bruce [25] with minor modifications. Both the cauda epidydimis were dissected out and placed in 1 mL NaCl (0.9 %). Tissue debris was removed by passing the suspension in two layers of muslin cloth. The sperms were released by mechanical disruption and washing of the epididymis. A drop of the cell suspension was smeared on clean and grease free slide, air dried and fixed in methanol for 10 min. The slides were stained in 2 % Eosin and 1000 sperms were scored per animal to estimate the frequency of sperms with abnormal head morphology. Various types of SHA such as amorphous, beaked, hooked, hook less, dwarf etc. were recorded. Epididymal sperm count was done using Neubauer’s hemocytometer. The cell number was multiplied by × 10^6^ and the sperm number was expressed as cells per epididymis.

### Biochemical estimations

Estimation of lipid peroxidation in tissues of liver, kidney and intestine was done by measuring the thiobarbituric acid reacting substance (TBARS) and was expressed in terms of malonaldehyde (MDA) content [26]. Briefly, the tissue homogenate (10 %) was prepared in ice-cold normal saline and centrifuged for 10 min at 3000 rpm. One milliliter of the supernatant was incubated at 37 ± 0.5 °C for 2 hrs. To each sample 1 mL of trichloroacetic acid (10 %) was added, mixed properly and centrifuged at 2000 rpm for 5 min. at 4 °C. An equal volume of thiobarbituric acid (0.67 %) was added to the reaction mixture, mixed properly and kept in boiling water bath for 10 min. The samples were then cooled, diluted with 1 mL of distilled water and optical density was measured at 535 nm in a Genesys-20 (Thermo Scientific, USA) spectrophotometer. The final MDA values were expressed as nmol/g wet tissue.

GSH levels in the tissues were estimated using 5,5’-dithiobis-2-nitrobenzoic acid (DTNB) method [27]. Tissues were homogenized in 5-8 mL of EDTA (0.02 M). It was then diluted with 2 mL of ice-cold distilled water. Then 1 mL of trichloroacetic acid (50 %) was added and mixed properly followed by centrifugation at 6000 rpm for 15 min. The supernatant (1 mL) was mixed with 2 mL of Tris buffer (0.4 M; pH 8.9) and DTNB (0.1 M). The absorbance was read at 410 nm and results were expressed in terms of μmol/g wet tissue.

SOD activity in the tissues was evaluated following the method of Marklund and Marklund [28]. Tissue homogenate (10 %) was prepared and pyragallol was used for SOD estimation. Absorbance was read at 420 nm at 1 min intervals for 3 min. The final calculated SOD values were expressed as units/ gram wet tissue.

### Statistical analysis

All the quantitative data were expressed as mean ± SE and one-way analysis of variance (ANOVA) was used to determine the significance of the parameters and Post hoc analysis was performed for multiple comparisons among the treatment groups using Tukey’s multiple comparison tests. Significance was accepted at ‘*P’* value less than 0.05. The analyses were performed using GraphPad Prism version 4.0 for Windows, GraphPad Software, San Diego California USA.

## Results

### Micronucleus assay

**Fig. 1:**
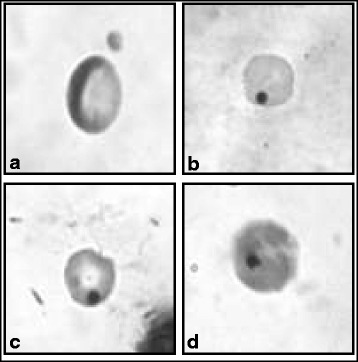
The photomicrographs showing normal (**a**), micronucleated (**b** and **c**) polychromatic, and (**d**) micronucleated normochromatic erythrocyte

Genotoxicity of SA and SG extract alone or in combination were determined by using bone marrow MN assay. Percentage of micronucleated PCEs (Fig. 1) and PCE/NCE ratio (Table 1) were evaluated. It was observed that there was a statistically significant increase in micronucleated PCEs in all the treated groups as compared to the control group (Table 1). There was a dose dependent increase in percentage of MN in SA treated groups and groups receiving both SA and SG extract. However, any additive effect of SA + SG extract as compared to only SA or only SG was not observed in the tested dose range.

**Table 1:** Frequency of micronucleus (MN) in mice bone marrow PCEs cells and PCE/NCE ratio induced by arsenic and sadagura alone and in combination

Treatment groups	Total PCE /n	% PCE with MN (mean ± S.E)	Total NCE	PCE/NCE (mean ± S.E)
CON	12000/6	0.06 ± 0.03	5098	2.39 ± 0.13
SA1	12000/6	0.23 ± 0.04^a*^	5044	2.40 ± 0.11
SA2	12000/6	0.43 ± 0.04^a***^	5160	2.34 ± 0.10^c^
SG	12000/6	0.22 ± 0.02^b*^	4760	2.52 ± 0.06^d^
SA1 + SG	12000/6	0.33 ± 0.01^***^	5334	2.26 ± 0.06
SA2 + SG	12000/6	0.45 ± 0.02^b***^	6625	1.86 ± 0.13^cd*^

Description of treatment groups is given in methodology section. *PCE* Polychromatic erythrocyte, *NCE* Normochromatic erythrocyte. Statistical analysis: ANOVA. Groups bearing the same superscript are significantly different from each other: a, b = p < 0.001; d = p < 0.01; c = p < 0.05. Values are significantly different from control: * = p < 0.05; ** = p < 0.01; *** = p < 0.001

The treatments differentially affected the PCE/NCE ratios. Statistically significant decrease in PCE/NCE ratio (*p* < 0.05) (Table 1) was observed only in highest combined dose of SA and SG as compared to control. Besides, there was statistically significant decrease in PCE/ NCE ratio between the highest combined dose and their individual counterparts SA *p* < 0.05 (SA2 v/s SA2 + SG) and SG *p* < 0.01 (SG v/s SA2 + SG) respectively (Table 1).

### Sperm head abnormality assay and sperm count

**Fig. 2:**
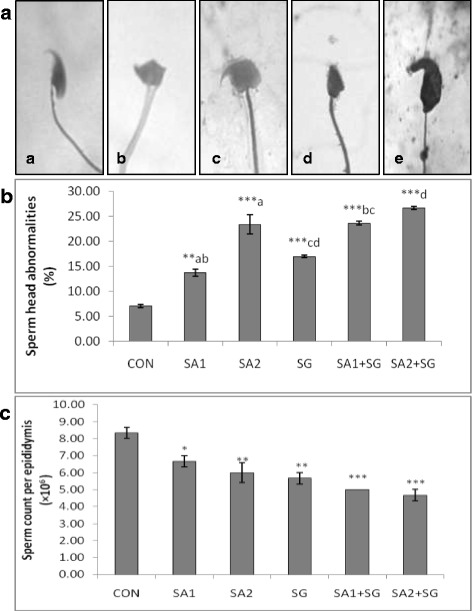
**a**: Representative photomicrographs showing normal (**a**), and abnormal (*b-e*), sperm head morphologies observed, **b**: Histogram showing percentage of sperm head abnormalities and **c**: Histogram showing sperm count per epididymis following treatment in different experimental groups. Values are significantly different from the control group: *p* < 0.05 (*); *p* < 0.01(**); *p* < 0.001(***). Values sharing similar letters are significantly different from each other: *p* < 0.001(*a*, *b*, *d* ); *p* < 0.01(**c**). Description about the treatment groups is given in the methodology section

After the exposure period, different types of sperm head morphology namely amorphous, beaked, hook less, pinheaded, double head, banana shaped, giant and dwarf were observed (Fig. 2a). There was a notable dose dependent increase in SHA in all the treated groups as compared to the control (Fig. 2b). Both SA and SG alone and in combined showed statistically significant impairment of SHA (*p* < 0.001) when compared to the control, suggesting role of the test substances in male germ cell toxicity. Sperms with ‘amorphous’ head morphology were the most frequently observed while ‘giant’ shaped sperm head morphology were the least observed. Statistically significant increase in SHA in SA1 + SG group was observed as compared to only SA1 or SG groups. There was no significant difference in SHA in SA2 + SG as compared to only SA2 or SG. A dose dependent decline in the total sperm count was observed in all the treatment groups as compared to the control (Fig. 2c). However, there was no significant difference in the sperm count among the treatment groups themselves.

### Changes in lipid peroxidation level

**Fig. 3:**
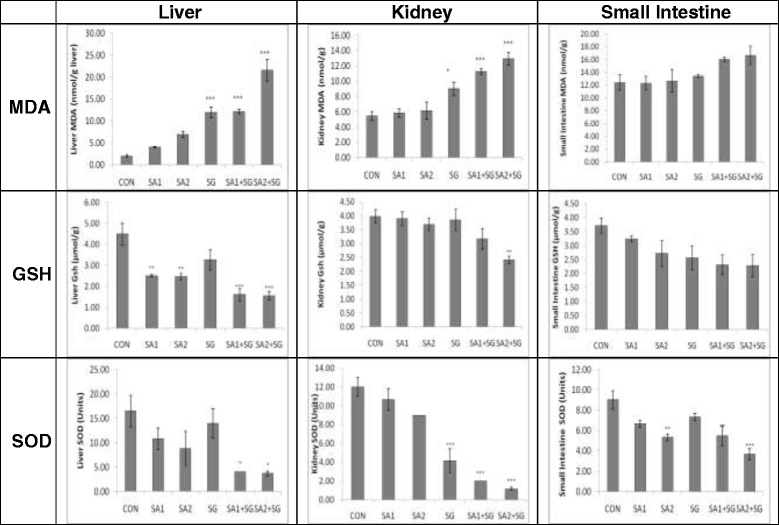
Histograms show MDA, GSH and SOD levels in different tissues. Values are significantly different from the control: *p* < 0.05 (*); *p* < 0.01(**); *p* < 0.001(***). Description about the treatment groups is given in the methodology section

There was a gradual increase in the hepatic MDA concentration from control group to the highest combined treated group. SG, SA1 + SG and SA2 + SG showed increased MDA levels which were 11.97 ± 1.23, 12.14 ± 0.51 and 21.55 ± 2.50 nmol MDA/g wet tissue respectively as compared to control value of 2.00 ± 0.32 nmol MDA/g wet tissue (*p* < 0.001) (Fig. 3; Table 2). Both the tested doses of SA showed increase in the MDA level as compared to control, but this was not statistically significant. This observation indicates that SG alone and in combination with SA induces more lipid peroxidation in liver than only SA.

**Table 2:** Post hoc analysis showing p value at different treatment groups and tissues with respect to MDA, GSH and SOD levels following 90 days of treatment

	MDA			GSH			SOD		
	Liver	Kidney	Intestine	Liver	Kidney	Intestine	Liver	Kidney	Intestine
SA1 vs SA2	NS	NS	NS	NS	NS	NS	NS	NS	NS
SA1 vs SA1 + SG	*p* < 0.001	*p* < 0.001	NS	NS	NS	NS	NS	*p* < 0.001	*p* < 0.05
SA2 vs SA2 + SG	*p* < 0.001	*p* < 0.001	NS	NS	*p* < 0.05	NS	NS	*p* < 0.001	NS
SG vs SA1 + SG	NS	NS	NS	*p* < 0.05	NS	NS	NS	NS	NS
SG vs SA2 + SG	*p* < 0.001	*p* < 0.05	NS	*p* < 0.05	*p* < 0.05	NS	NS	NS	*p* < 0.001
SA1+ SG vs SA2 + SG	*p* < 0.001	NS	NS	NS	NS	NS	NS	NS	NS

*SA1* Sodium arsenite 0.2 mg/kg body weight/da), *SA2* Sodium arsenite 2 mg/kg body weight/day, *SG Sadagura* extract 5mg/kg body weight/day, *n* number of animals, *MDA* Malonaldehyde, *GSH* Glutathione, *SOD* Superoxide dismutase

Compared to the control group (5.42 ± 0.59 nmol MDA/g wet tissue) the renal levels of MDA significantly increased in SG (*p* < 0.05) as well as in both the combined treatment groups (*p* < 0.001) (Table 2; Fig. 3). In SG, SA1 + SG and SA2 + SG groups, the MDA level was found to be 8.99 ± 0.86, 11.25 ± 0.37 and 12.93 ± 0.83 nmol MDA/g wet tissue respectively . A similar observation like that of liver MDA was observed in the kidney tissue. In the intestinal tissue, MDA level was higher in both the combined groups (16.02 ± 0.36 and 16.66 ± 1.40 nmol MDA/g wet tissue in SA1 + SG and SA2 + SG, respectively) when compared to the control (12.39 ± 1.22); but these were not statistically significant suggesting minimal lipid peroxidation by the test chemicals on the intestinal tissue (Fig. 3).

### Changes in reduced glutathione levels

When compared with the control (4.50 ± 0.52 μmol/g wet tissue), both the combined (*p* < 0.001) as well arsenic treated groups (*p* < 0.01) showed a significant decrease in hepatic GSH levels (Fig. 3). In SA1, SA2, SA1 + SG and SA2 + SG the GSH values were 2.50 ± 0.06, 2.46 ± 0.16, 1.61 ± 0.29 and 1.55 ± 0.21 μmol/g wet tissue respectively. Comparative analysis between the groups showed that the GSH content in SG treated group was significantly different from SA2 + SG (*p* < 0.05) (Table 2).

In kidney tissues the highest combined treated group (SA2 + SG) showed a significant decrease (*p* < 0.01) in GSH level as compared to the control group (Fig. 3). Comparative analysis among the treatment groups showed that SG and SA in higher dose (*p* < 0.05) and SA in lower dose (*p* < 0.01) treated groups were significantly different when compared to higher combined treatment group of SA2 and SG (Table 2). There was a dose dependent decrease in intestinal GSH values from control to the higher combined treatment groups, although this was not statistically significant (Fig. 3). The GSH value in the control group was 3.71 ± 0.27 μmol/g wet of intestinal tissues.

### Changes in superoxide dismutase activity

Hepatic SOD activity in both the combined groups were significantly lower (*p* < 0.05) as compared to the control group. SA1, SA2 and SG showed lower SOD activity but were not statistically different from control (Fig. 3). The SOD values in control, SA1, SA2, SG, SA1 + SG and SA2 + SG were 16.50 ± 3.23, 10.83 ± 2.21, 8.83 ± 3.46, 14.00 ± 3.03, 4.00 ± 0.00 and 3.67 ± 0.49 units respectively. However, this decrease was not statistically significant compared to the control (Table 2).

The kidney tissue of mice treated with only SG and both the combined treatment groups had a significantly lower (*p* < 0.001) SOD activity as compared to the control group (Fig. 3). Post hoc analysis showed a significant decrease in SOD level in combination treatment groups as compared to their arsenic counterparts (*p* < 0.001) ( Table 2). In intestinal samples, SA2 and SA1 + SG (*p* < 0.01) as well as SA2 + SG (*p* < 0.001) showed a significant lower SOD activity as compared to the control group (Fig. 3). Post hoc and Tukey’s multiple comparison test showed significant difference between SA1 and SA1 + SG as well as between SG and SA2 + SG (*p* < 0.001) groups (Table 2).

## Discussion

Our study indicates that SA as well as SG has the potential to cause genotoxicity in mouse none marrow cells. Long-term exposure to SA alone as well as in combination with SG induced significantly higher micronucleus formation as compared to the control. Previous studies have reported the genotoxic potential of arsenite [29, 30]. Hepatic methyl donor status may affect arsenic related genotoxicity [31]. There are reports of chromosomal aberrations and chromosomal rearrangements in bone marrow cells of mice induced by arsenic [32, 33]. However, there is no previous report on SA and SG or any form of smokeless tobacco combined genotoxicity in mice test system. SG is one of the most prevalent smokeless tobacco in Southern Assam and is often chewed with betel quid or areca nut. There are limited literature available on SG and its association with the DNA damage. There are many types of the tobacco-areca nut specific nitrosamines that have been detected in the saliva of chewers [34] and some of these are potent mutagens and carcinogens [35]. Nicotine, which is a major alkaloid present in the tobacco leaves, is a weak clastogen [36].

In our study, a significant increase in SHA and reduction in epididymal sperm count as compared to control group were among the other long-term effects of SA and SG exposure. Sperm head abnormal morphology may arise due to various mechanisms. There may be alteration in testicular DNA [37], chromosomal aberrations which may occur during the packaging of genetic material in the sperm head or due to incidence of point mutation in testicular DNA [38]. Exposure of chemicals could produce hormonal changes causing disturbances in normal spermatogenesis or could cause abnormalities in the seminal fluid leading to structural or functional impairment of the sperms. [39] Long-term SA treatment leads to decrease in absolute and relative testicular weight and decrease in the activity of 17 β-HSD along with increase in LDH, γGT activity [40]. Studies have shown that long-term exposure to sodium meta-arsenite leads to reduction in seminiferous tubular diameter and various gametogenic cell populations as well increase in leydig cell atrophy [41].

Our study indicates that long-term exposure to SA and SG for 90 days in mice leads to a substantial increase in oxidative stress in liver, kidney and intestine. Figure 3 shows the variations in the different oxidative stress parameters in different tissues studied. Liver is the primary detoxifying organ of the body. Long-term exposure to arsenic may lead to severe fibrosis in the liver cells. Histological studies have shown various alterations like inflammatory cell infiltration, hepatocyte vacuolization and parenchyma disorganization in the liver, and tubular epithelium vacuolation and interstitial blood in the kidneys of arsenic treated mice and subsequent reversal of the toxicity and signs of protection by administration of antioxidants [42]. Chronic administration of arsenic has been variously reported to induce lipid peroxidation and inhibition of superoxide dismutase in different tissues in mice. Epithelial cells of the jejunum and the colon in mice are highly dependent on GSH, and GSH deficiency leads to marked cellular degeneration [43].

## Conclusion

The present findings suggest that induction of MN in the bone marrow cells and impairment of the sperm head morphology could possibly involve a common mechanism in the form of oxidative stress induced by SA and SG. Arsenic and SG have the potential to cross the testis-blood barrier and cause male germ cell toxicity. There was a progressive increase in MDA level and decline in the GSH and SOD levels in all the three tissue tested as compared to the control group. This indicates that ROS generation possibly plays a role in SA and tobacco mediated genotoxicity and male germ cell toxicity. However, in the combined treatment of SA and SG the lack of additive effects on micronucleated PCEs, SHA and total sperm count suggest that people consuming smokeless tobacco and residing in arsenic contaminated areas may not be at additional risk of genotoxicity and decline in sperm count apart form the risks arising out of the individual components separately. However, as they can individually induce MN and SHA and lower sperm count, consumption of SG should be avoided at all cost and arsenic mitigation strategy should be initiated in the affected areas.
